# Lateral- or prone-position video-assisted thoracic surgery for dumbbell-type posterior mediastinal tumors: pros and cons

**DOI:** 10.1007/s12055-022-01343-0

**Published:** 2022-03-28

**Authors:** Yuki Matsumura, Hikaru Yamaguchi, Kazuyuki Watanabe, Hiroyuki Suzuki

**Affiliations:** 1 Department of Chest Surgery, Fukushima Medical University, 1 Hikarigaoka, Fukushima, Japan; 2 Department of Orthopaedic Surgery, Fukushima Medical University, Fukushima, Japan

**Keywords:** Posterior mediastinal tumor, Dumbbell type, Video-assisted thoracic surgery

## Abstract

Surgery for dumbbell-type posterior mediastinal tumors (D-PMTs) is difficult because surgeons should confirm the tumor’s extension into the spinal cord and pay attention to the Adamkiewicz artery. We describe two patients of D-PMTs who underwent lateral- or prone-position video-assisted thoracic surgery (VATS). In patient 1 (a 70-year-old woman), the tumor extended to the spinal canal through the fourth thoracic intervertebral foramen. After hemi-laminectomies, she was moved to the lateral position, and the tumor was resected. In patient 2 (a 16-year-old boy), the tumor extended to the spinal canal through the seventh thoracic intervertebral foramen. Additionally, 320-row high-resolution computed tomography showed Adamkiewicz arteries running through the sixth and eighth thoracic intervertebral foramina. After laminectomy, the tumor was resected without repositioning. Prone-position VATS is a useful approach for D-PMTs because it provides a better view of the vertebrae compared with the lateral position. We discuss the advantages and disadvantages of both approaches.

**Supplementary Information:**

The online version contains supplementary material available at 10.1007/s12055-022-01343-0.

## Introduction

Among mediastinal tumors, dumbbell-type posterior mediastinal tumors (D-PMTs) are difficult to treat surgically. There are three reasons for this difficulty. First, surgeons must confirm the tumor’s extension into the spinal cavity. Second, the surgical field is always deep around the vertebral body, and the aorta and lung often interfere with the visual field and forceps manipulation. Third, surgeons must pay attention to the Adamkiewicz artery (AKA). We describe two patients with D-PMTs that were resected by lateral- or prone-position video-assisted thoracic surgery (VATS) after orthopedic laminectomy, and we discuss the advantages and disadvantages of each approach.

## Case reports

**Fig. 1:**
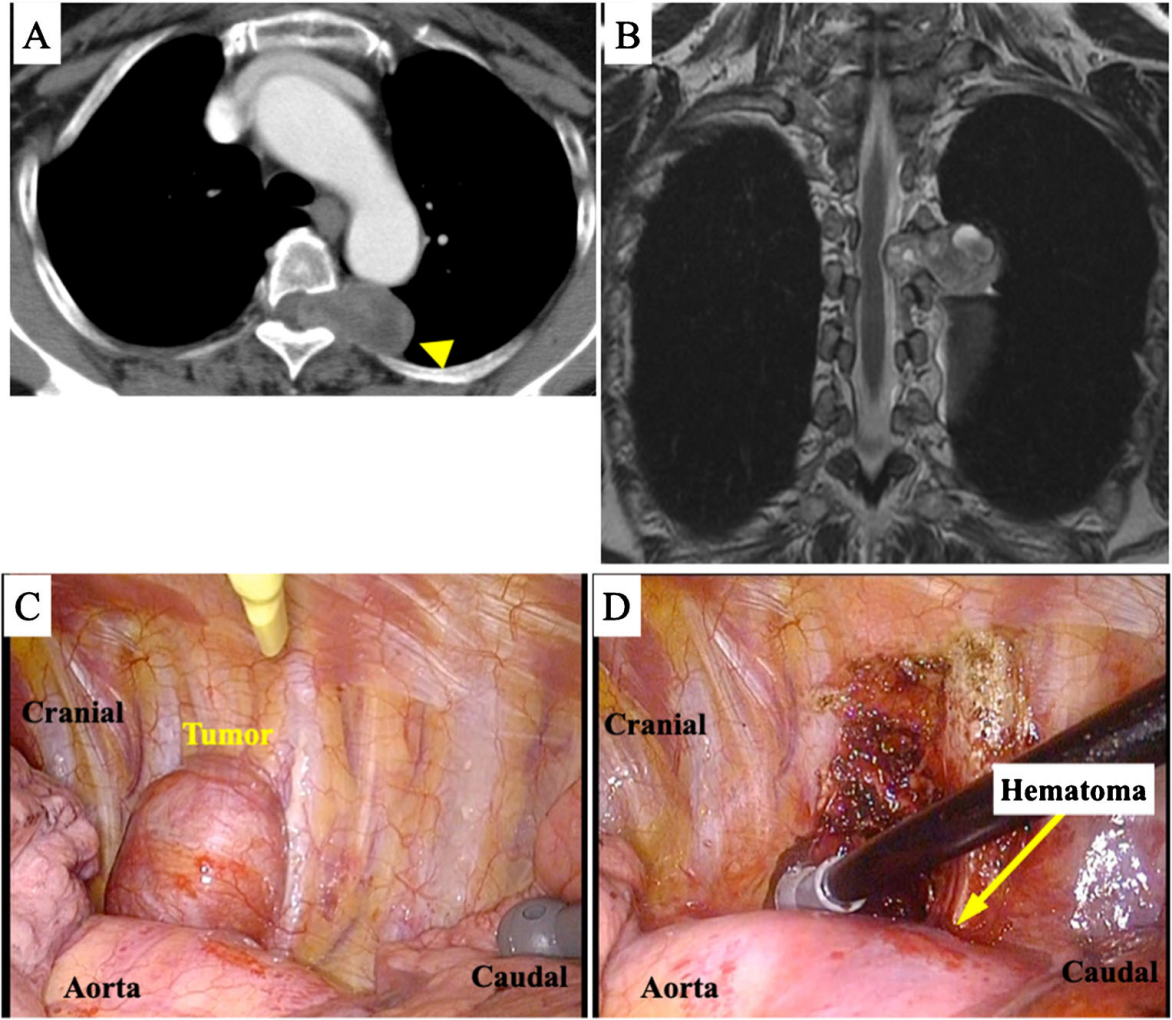
Images of patient 1. **A** and **B** The tumor was located in the left posterior mediastinum and was suspected to extend to the spinal canal through the fourth thoracic (Th4) intervertebral foramen on computed tomography (CT) and magnetic resonance imaging (MRI). **C** and **D** Intraoperative photographs. The descending aorta concealed the vertebral side of the tumor. The forceps contacted the aorta, which resulted in a pleural hematoma

Patient 1: A 70-year-old Japanese woman had a tumor in the left posterior mediastinum that extended to the spinal canal through the fourth thoracic (Th4) intervertebral foramen on computed tomography (CT) and magnetic resonance imaging (MRI) (Fig. 1A, B). Hemi-laminectomies of Th3–5 and left facetectomy of Th4 and Th5 were performed, and the spinal dura mater was opened. The posterior branch of the Th4 spinal nerve was ligated and dissected (Eden type 2). The patient was then repositioned to the right lateral position, and the tumor was resected by VATS. The descending aorta concealed the vertebral side of the tumor (Fig. 1C and the Video), which forced the surgeons to retract the aorta. Additionally, the forceps contacted the aorta and created a pleural hematoma (Fig. 1D). Pathology indicated a schwannoma.

**Fig. 2:**
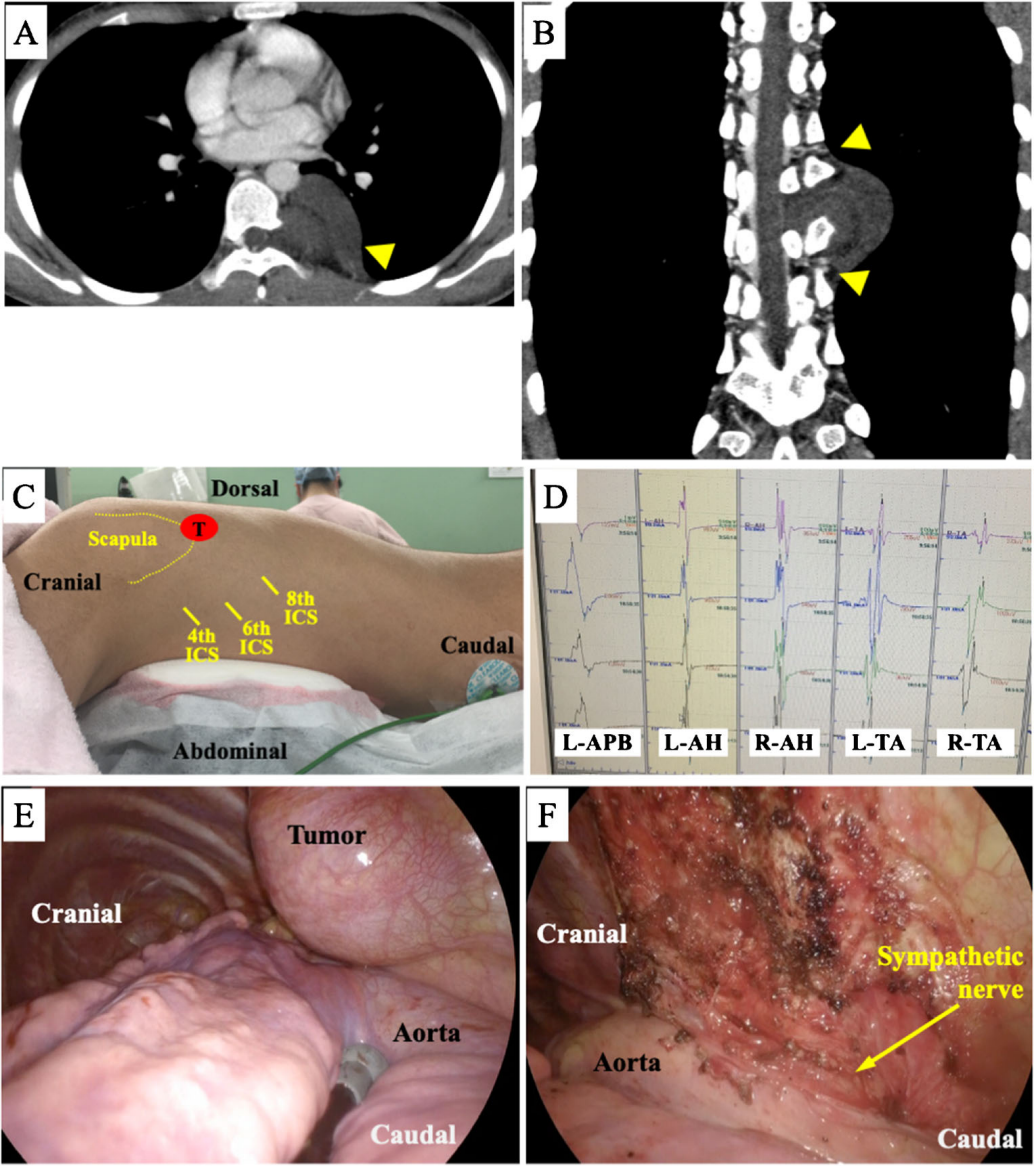
Images of patient 2. **A** The tumor was located in the left posterior mediastinum and was suspected to extend to the spinal canal through the seventh thoracic (Th7) intervertebral foramen on computed tomography (CT). **B** The AKA ran through the sixth thoracic (Th6) and eighth thoracic (Th8) intervertebral foramina (arrowheads). **C** Prone position during VATS and the locations of the three thoracic ports. These positions did not change during the operation. **D** Motor evoked potentials (MEPs) were used intraoperatively to monitor the descending motor pathway. Intraoperative deterioration of MEPs was not observed during the operation. *L* left, *R* right, *APB* abductor pollicis brevis, *AH* abductor hallucis, *TA* tibialis anterior. **E** and **F** Intraoperative photographs showing that both the lung and aorta descended anteriorly with gravity, which produced good surgical fields and kept the forceps from contacting the aorta

Patient 2: A 16-year-old Japanese boy had a tumor in the left posterior mediastinum that extended to the spinal canal through the Th7 intervertebral foramen on CT and MRI (Fig. 2A). High-resolution CT showed AKAs running through the Th6 and Th8 intervertebral foramina (Fig. 2B). Laminectomy and left facetectomy of Th7 and Th8 were performed, and the tumor was detached from the spinal dura mater with ligation of the Th7 spinal nerve (Eden type 3). The tumor was then resected by VATS without repositioning (Fig. 2C). Intraoperatively, the descending motor pathways were monitored by motor evoked potentials (MEPs; Fig. 2D). During VATS, both the lung and aorta descended anteriorly with gravity, which produced good surgical fields and prevented the forceps from contacting the aorta (Fig. 2E, F). Intraoperative deterioration of MEPs was not observed, and no postoperative paraplegia occurred. Pathology indicated a neurofibroma.

## Discussion

A larger part of D-PMTs is located on the vertebral side than on the descending aorta side, and maintaining a good surgical field is difficult. McKenna et al. first reported the use of VATS to treat D-PMT in the prone position. This procedure is unpopular probably because of the technical difficulties of VATS in the prone position [1]. However, this approach is being adopted more often for esophageal cancer because of its minimal invasiveness and good surgical view [2, 3]. Recent studies have also described robotic resection of thoracic dumbbell-type tumors without repositioning from the prone position and have claimed the benefits of the prone position [4, 5]. We have performed both lateral- and prone-position VATS for D-PMTs and considered the advantages and disadvantages of each approach. Regarding the intraoperative visual field, the tumor was located behind the aorta in the lateral position (Fig. 1C), which forced the operators to retract the aorta to expose and dissect the vertebral side of the tumor. In contrast, in the prone position, the lung and aorta both descended anteriorly with gravity (Fig. 2E). Therefore, the operators did not need to retract organs to gain a better visual field of the tumor’s vertebral side [6]. Regarding forceps operability, the forceps contacted the aorta and created a pleural hematoma in the lateral position (Fig. 1D); this accidental event did not occur in the prone position. However, more of the tumor was located on the upper (ceiling) side of the screen in prone-position VATS. Therefore, the thoracoscopic ports should be placed on the anterior side of the scapula or on the mid-axillary line (Fig. 2C), which may be less familiar to general thoracic surgeons. Regarding the AKA, the prone position allowed the operators to detect the roots of the intercostal arteries more easily than in the lateral position because the patient’s sympathetic nerves and intervertebral foramina were well exposed (Fig. 2F). Prone-position VATS can also shorten the operation time because repositioning is not required and this position decreases the reposition-related risks of anesthetic complications, such as migration of the endotracheal tube or adverse effects on respiration and circulation. A possible disadvantage of prone-position VATS is that repositioning to the lateral position may be necessary when open thoracotomy is required in cases of major bleeding. During anesthesia, the prone position involves the possibility of injuring the skin, eyes, and peripheral nerves owing to the effects of body pressure. Fortunately, this position has recently become popular, especially in esophageal surgery; therefore, anesthesiologists are becoming accustomed to differential lung ventilation in this position.

## Conclusion

We described two patients who underwent resection of D-PMTs by lateral- or prone-position VATS with orthopedic laminectomy. Prone-position VATS is considered a safe and superior approach for D-PMTs.

## Supplementary information

ESM 1 (MP4 52.4 mb)
